# Plasma Lipid Profile and Systemic Inflammation in Patients With Cancer Cachexia

**DOI:** 10.3389/fnut.2020.00004

**Published:** 2020-01-31

**Authors:** Daniela Mendes dos Reis Riccardi, Rodrigo Xavier das Neves, Emidio Marques de Matos-Neto, Rodolfo Gonzalez Camargo, Joanna Darck Carola Correia Lima, Katrin Radloff, Michele Joana Alves, Raquel Galvão Figuerêdo Costa, Flávio Tokeshi, José Pinhata Otoch, Linda Ferreira Maximiano, Paulo Sérgio Martins de Alcantara, Alison Colquhoun, Alessandro Laviano, Marilia Seelaender

**Affiliations:** ^1^Cancer Metabolism Research Group, Institute of Biomedical Sciences University of São Paulo, São Paulo, Brazil; ^2^Laboratory of Integrative Cancer Immunology, Center for Cancer Research, National Cancer Institute, NIH, Bethesda, MD, United States; ^3^Department of Physical Education, Federal University of Piaui, Teresina, Brazil; ^4^University Hospital of the University of São Paulo, São Paulo, Brazil; ^5^University of São Paulo Medical School (FMUSP), São Paulo, Brazil; ^6^Department of Cell and Developmental Biology, Institute of Biomedical Sciences University of São Paulo, São Paulo, Brazil; ^7^Department of Clinical Medicine, Sapienza University of Rome, Rome, Italy

**Keywords:** cancer cachexia, inflammation, free-fatty acids, lipid profile, cytokines

## Abstract

Cancer cachexia affects about 80% of advanced cancer patients, it is linked to poor prognosis and to date, there is no efficient treatment or cure. The syndrome leads to progressive involuntary loss of muscle and fat mass induced by systemic inflammatory processes. The role of the white adipose tissue (WAT) in the onset and manifestation of cancer cachexia gained importance during the last decade. WAT wasting is not only characterized by increased lipolysis and release of free fatty acids (FFA), but in addition, owing to its high capacity to produce a variety of inflammatory factors. The aim of this study was to characterize plasma lipid profile of cachectic patients and to correlate the FA composition with circulating inflammatory markers; finally, we sought to establish whether the fatty acids released by adipocytes trigger and/or contribute to local and systemic inflammation in cachexia. The study selected 65 patients further divided into 3 groups: control (N); weight stable cancer (WSC); and cachectic cancer (CC). The plasma FA profile was significantly different among the groups and was positively correlated with pro-inflammatory cytokines expression in the CC patients. Therefore, we propose that saturated to unsaturated FFA ratio may serve as a means of detecting cachexia.

## Introduction

Cancer is nowadays recognized as the second leading cause of death worldwide according to the World Health Organization (WHO). Approximately 80% of patients with advanced cancer suffer from cachexia, a syndrome directly responsible for about 22–40% of all the deaths attributed to the disease (1, 2). Cachexia is defined as a multifactorial syndrome, in which there is progressive and involuntary loss of body mass (often accompanied by fat mass loss), which cannot be completely reversed by conventional nutritional therapy, leading to progressive wasting of the body energy stores (3). Despite being a huge health problem of highest clinical importance, the syndrome is very often underdiagnosed and consequently, rarely therapeutically targeted (1, 4, 5). There is growing evidence that systemic inflammation is a major player in the development and progression of cancer cachexia. Within the tumor microenvironment, cancer cells and interacting host cells produce a battery of pro-inflammatory cytokines, such as tumor necrosis factor alpha (TNF-α) and Interleukin 6 (IL-6), among many others, that play a central role in the induction and in the course of cancer cachexia (6–8). Chemokines, which are produced in the different body compartments, provoke immune cell infiltration, and play a crucial role in fat depletion and local adipose tissue inflammation (9, 10). The WAT is also an active player in metabolism regulation via adipokines secretion along with physiological and pathological processes, including the regulation of immunity, and inflammation (11). WAT secrets more than 100 pro- and anti-inflammatory factors (9, 10). Studies of our research group (12–16) showed that actually, in both animals and patients, WAT is a major source of inflammatory factors in cachexia and therefore, a possible contributor to the generation and maintenance of the systemic inflammation that is associated with the syndrome. Adipose tissue inflammation is concomitant to lipolysis, since the regulating enzymes hormone-sensitive lipase (HSL) and triacylglycerol lipase (ATGL) are responsive to pro-inflammatory cytokines such as IL-1β, IL- 6, TNF-α, and IFN-γ (9, 17). The result of exacerbated lipolysis in cachexia is the release of free fatty acids (FFA). FFA themselves present inflammatory properties, and the FFA released from adipocytes are capable of activating macrophages (18). Saturated fatty acids (SFA) act as ligands of receptors related to the innate immune response, such as the Toll-like receptor type four (TLR-4), resulting in increased production of pro- inflammatory cytokines (19, 20). Endogenous or dietary polyunsaturated fatty acids (PUFA) are precursors for pro- and anti-inflammatory lipid mediators (21). While PUFA of the omega-6 family (n-6) are related to the formation of pro-inflammatory factors such as prostaglandins or leukotrienes (22), Omega-3 PUFAs (n-3) are precursors for anti-inflammatory lipid mediators, namely lipoxins or resolvins (21). N-3 PUFAs are widely associated with anti-inflammatory effects in chronic inflammatory conditions, including rheumatoid arthritis, and Crohn's disease. Moreover, Murphy et al. evaluated the FA profile in gastrointestinal cancer patients and found a correlation between the time to death of the patient and the plasma content of n-3 and n-6 PUFAs (23). The fatty acid profile and its impact on inflammatory processes is intensively studied in obesity (24–27). However, little is known about the specific types of fatty acids released during human cancer cachexia and how the fatty acid profile could contribute to the progression of the systemic inflammation. Therefore, this study not only investigated alterations of the fatty acid profile in cancer cachexia, but also explored the ability of different types of fatty acids to correlate with inflammation. To this end, we assessed and compared the proportion of circulating fatty acids and inflammatory cytokines in cachectic cancer patients with that of non-cachectic cancer and control patients.

## Materials and Methods

### Ethics Committee Approval

This study was carried out in accordance with the recommendations of Ethics Committee on Research involving Human Subjects of the Biomedical Sciences Institute/University of São Paulo (1117/CEP), the Human Ethics Committee of the University Hospital/University of São Paulo (CEP 1388/14) and registered at the Brazilian platform for human studies under the CAEE number 14197413.9.0000.5467. All subjects gave written informed consent in accordance with the Declaration of Helsinki. The protocol was approved by the previously mentioned committees.

### Patient Recruitment

Adult patients of both genders (*n* = 65; age range: 19–82 years) attending the Surgical Clinic of the University Hospital/ University of São Paulo for gastrointestinal cancer or for non-malignant diseases (i.e., hernia or cholecystectomy) were considered for the study. Exclusion criteria included: delivery of chemotherapy at the time of the study or chronic anti-inflammatory treatment; renal, or hepatic failure, AIDS, inflammatory bowel disease or other chronic inflammatory diseases not related to cachexia, such as obesity or autoimmune disorders, all diagnosed by the physicians involved in the project. Control patients who presented serum C-reactive protein (CRP) concentration >5 mg/L were also excluded, due to the presence of inflammation (28). After applying all exclusion criteria our study ended up with 65 patients and were signed up into the following: control group (*N*; *n* = 26)—patients that underwent surgical removal of hernia or cholecystectomy; and weight stable (WSC; *n* = 16) or cachectic cancer (CC; *n* = 23)—groups of patients subjected to gastrointestinal tumor excision surgery. After obtaining the informed consent, blood samples were collected just before the surgical procedure.

### Cachexia Diagnosis

Cachexia was diagnosed by the criteria proposed by Evans et al. (1). Involuntary body weight loss in the past 6 months ≥5%; body mass index (BMI) <20 kg/m2 for patients younger than 65 years and <22 kg/m^2^ for patients older than or equal to 65 years; plasma albumin concentration <3.2 g/dL; evidence of inflammation (high plasma concentration of CRP>5 mg/L); low scores of the QLQ-C30 and FAACT-ESPEN questionnaires—these questionnaires included three topics: anorexia, fatigue and quality of life (29).

### Blood Collection and Biochemical Parameters Assessment

Approximately 20 mL of blood were collected prior to the surgical procedure, requiring patient-fasted overnight (8–12 h) before surgery, by a trained health professional. Blood was placed in tubes with and without anticoagulant (EDTA) and then centrifuged at 3,000 rpm for 15 min at 4°C to obtain plasma and serum, respectively. Then, plasma and serum were transferred to plastic microtubes and stored at −80°C for analysis. The biochemical analyses for hemoglobin, C-reactive protein, albumin, glucose, total cholesterol, low density lipoprotein (LDL), high density lipoproteins (HDL), and triglyceride were performed using LABMAX 240® equipment, employing commercial kits (Labtest Diagnóstica SA, Lagoa Santa, Brazil). NEFA was performed employing NEFA kit (Wako Chemicals, USA) following the manufacturer's instructions, and glycerol was performed employing Free Glycerol Reagent kit (Sigma-Aldrich Brazil Ltda.) following the manufacturer's instructions. Plasma pro and anti-inflammatory cytokines content measurement (CCL2, IL-1β, IL-6, IFN-γ, TNF-α, IL-10, IL-8, IL-4, IL-15, and IL-1ra).

The assessment of cytokines in plasma were carried out according the manufacturer's instructions of the HCYTMAG-60K-PX30 human cytokine panel. In brief, undiluted plasma samples were incubated with MagPlex® beads coated with specific antibodies for 2 h. The antigens were detected using secondary biotinylated streptavidin labeled phycoerithrin capture antibodies. The intensity of phycoerithrin was measured with the Luminex instrument MAGPIX (Life Technologies) and processed with the Analyst 5.1 software. The cytokine contents were then normalized to total protein concentration.

### Identification of Fatty Acids by Gas Chromatography and Quantification by Mass Spectrometry (GC-MS)

For the analysis of the plasma fatty acid profile, the fatty acid fraction was extracted with chloroform/methanol (30). Prior to GC-MS analysis, the fatty acid methyl esters were generated by heating up the lipid extracts in anhydrous methanol/sulfuric acid. The fatty acid methyl esters were then separated on a DB-23 column [(50% cyanopropyl) methyl polysiloxane 0.25 mm thick film, 0.250 mm; 60 m], with an injection inlet at 220°C, 250°C at the interface between the column and the mass spectrometer and helium carrier gas (99.999%) on a GC-MS (Shimadzu QP5050 model). The conditions of the experiment were programmed as follows: 150°C hold for 2 min post-injection (in the first 9 min the initial values have not been measured because the solvent may saturate the mass spectrometer), then 150–200°C at a rate of 10°C/min; 200–230°C at 1.3°C/min; 230–250°C at 10°C/min. The fatty acids were identified by comparison with retention times of authentic standards and by mass spectra as described by Ramos and Colquhoun (31).

### Statistical Analysis

Data were expressed as mean ± standard error or median [1st. quartile; 3rd. quartile]. The groups were compared using One-way ANOVA followed, as needed, for multiple comparisons by Tukey or Kruskal-Wallis method, if data presented non-parametric distribution. The Spearman correlation coefficient was obtained to evaluate the linear relationship between the variables of interest. The significance level was set at *p* < 0.05. For the analysis of qualitative parameters, location and tumor staging, the chi-square test was employed. All statistical procedures were performed with the assistance of the Statistics Sector of the Institute of Biomedical Sciences, USP, under the supervision of Mrs. Rosana Duarte Prisco. The statistical software Statgraphics® Centurion XVI version 2.16.04 (Statpoint Technologies, Inc. Warrenton, Virginia) was employed in the calculation.

## Results

### Cachexia Prevalence Was Higher in Advanced Cancer Stages and Associated With Lower Life Quality

The anthropometric characteristics (Table 1) showed no statistical differences in regard to patients' height and age, although patients in both cancer groups (WSC and CC), showed a tendency to be older than the controls (*p* = 0.057). Reported body weight 6 months prior to enrolment in the study was not altered among the groups but as expected, while current body weight of CC was significantly lower, when compared to WSC and N, the WSC group also exhibited lower current body weight compared to N (*p* < 0.05). However, statistically significant weight loss (> 10%) was exclusively observed in CC. In addition, body mass index (kg/m2) of CC, while >20 kg/m2, was significantly lower than in the control group and in WSC (~25 kg/m2). The overall quality of life, assessed with the QLQ-C30 questionnaire was significantly reduced (lower values = lower quality of life) in cachectic patients compared to patients in the other groups (Table 1). Furthermore, the final score of the FAACT-ESPEN-questionnaire evaluating the degree of anorexia also presented lower value (which means more anorexia) in CC (Table 1). These findings are consistent with the definition of cachexia employed to classify the groups (1). To investigate whether cachexia was associated with tumor stage chi-square test was employed, considering the grouped tumor stages I/II and III/IV. We identified a significantly larger proportion of patients with stage III/IV (69.6%, *p* = 0.003) in the cachectic group while the WSC group mainly consisted of patients of I/II stages (81.3%). However, the presence of cachexia did not correlate with tumor location (*p* = 0.736; Table 1).

**Table 1:** Clinical findings.

	***N***	**WSC**	**CC**	***p***
***N***	**26**	**16**	**23**	
Male/Female (*n*)	17/9 (65%/35%)	10/6 (62%/38%)	12/11 (52%/48%)	
Height (m)^a^	1.66 ± 0.02	1.62 ± 0.02	1.63 ± 0.02	0.394
Age (years)^a^	55.2 ± 2.49	64.8 ± 2.87	58.7 ± 2.61	0.057
Previous informed body mass (Kg)^a^	71.5 ± 2.9	70.1 ± 2.8	68.8 ± 2.7	0.721
Body mass (Kg)^a^	70.8 ± 2.7	65.6 ± 2.0^*^	58.7 ± 2.4^*^^#^	**<0.05**
Δ Body mass (Kg)^b^	0 [0; 0]	0 [−6.5; 0]	−10.0 [−13.0; −6.0]^*^^^#^^	**<0.001**
Δ Body mass (%)^b^	0 [0; 0]	0 [0; 9.5]	14.3 [8.8; 17.8]^*^^^#^^	**<0.001**
BMI (Kg/m^2^)^b^	25.0 [24.0; 27.4]	24.8 [22.4; 27.9]	21.3 [20.1; 25.2]^*^^^#^^	**0.004**
QLQ-C30^b^	60.8 [59.0; 64.4]	57.7 [52.0; 62.3]	41.5 [38.8; 43.0]^*^^^#^^	**<0.001**
FAACT-ESPEN^2^	38.0 [35.0; 40.0]	35.5 [32.0; 37.0]	31.0 [25.0; 35.0]^*^	**<0.001**
**Tumor Staging**				
I	–	6 (37.5%)	1 (4.34%)	**0.003**^$^
IIA/IIB/IIC	–	7 (43.8%)	6 (26.6%)	
I/II	–	**13 (81.3%)**	**7 (30.4%)**	
IIIA/IIIB/IIIC	–	2 (12.5%)	9 (39.1%)	
IVA/IVB	–	1 (6.2%)	7 (30.4%)	
III/IV	–	**3 (18.7%)**	**16 (69.6%)**	
**Tumor Site**				
Colon and rectum	–	11 (73.3%)	15 (68.2%)	0.736^&^
Stomach	–	4 (26.7%)	7 (31.8%)	

*^1^Data expressed as mean ± SEM. p = significance level of ANOVA*.

2 *Data expressed as median [1st quartile; 3rd quartile], p = significant difference by Kruskal-Wallis test: Δ the difference between the reported previous body mass and actual body weight; QLQ-C30 questionnaire of quality of life; FAACT-ESPEN, questionnaire to assess the degree of anorexia*.

* *Significant difference vs. N (p < 0.05)*.

# *Significant difference vs. WSC (p < 0.05)*.

$ *Significance level from chi- square test grouping stage I–II and III–IV*.

& *Significant difference in chi- square test*.

*p < 0.05 are highlighted in bold*.

### Alterations of Biochemical Serum Parameters and Inflammation in Cancer Cachexia

Considering the currently suggested biochemical parameters (1, 3) for the diagnosis of cachexia, we observed that hemoglobin in CC and WSC was actually lower when compared to N (Table 2), in addition we tested whether the CC is different compared to WSC and it showed a tendency to decease (0.065). Serum content of C-reactive protein, an important systemic inflammation marker, was about 10-fold higher in CC, compared to N and more than twice as high than in WSC (*p* < 0.001). The serum albumin content was significantly lower in CC (Table 2), when compared to N, however, the detected concentrations (4.03 [3.47; 4.59] g/dl) were superior to those published by the international consensus for the diagnosis of cachexia. When the individual plasma albumin concentration was analyzed, it was found that from 23 patients of the cachectic group, 19 showed albumin concentrations above 3.2 g/dL. Therefore, we decided to further assess the CRP/albumin ratio, having found higher values in the CC group, suggesting then that this ratio confirms the validity of the criteria that have been used by the Brazilian Consensus of Cachexia (32).

**Table 2:** Biochemical parameters of patients in each group.

	***N***	**WSC**	**CC**	***p***
Hemoglobin (g/dL)	14.7 [13.9; 15.8] (*n* = 23)	13.3 [11.7; 14.5]^*^ (*n* = 16)	11.3 [8.9; 12.9]^*^ (*n* = 20)	**<0.001**
CRP (mg/L)	1.15 [0.5; 3.4] (*n* = 26)	3.95 [1.1; 8.2] (*n* = 16)	10.95 [6.2; 12.2]^*^ (*n* = 22)	**<0.001**
Albumin (g/dL)	4.46 [4.14; 5.14] (*n* = 26)	4.54 [3.79; 4.93] (*n* = 16)	4.03 [3.47; 4.59]^*^ (*n* = 23)	**0.034**
CRP/Albumin (mg/g)	0.023 [0.012; 0.065] (*n* = 26)	0.093 [0.023; 0.230] (*n* = 16)	0.240 [0.135; 0.322]^*^ (*n* = 22)	**<0.001**

*Data are expressed as median [1st quartile; 3rd quartile], p = significance level Kruskal-Wallis test. CRP: C -reactive protein*.

* *Significant difference vs. N (p <0.05)*.

*^#^Significant difference vs. WSC (p < 0.05)*.

*p < 0.05 are highlighted in bold*.

### Changes in the Blood Lipid Profile During Human Cancer Cachexia

Lipoproteins, triacylglycerides (TAG) as well as total glycerol and FFA were measured and a significant reduction in HDL in CC compared to WSC was found (Table 3). The low density lipoprotein (LDL) fraction and total cholesterol were not altered among the groups. Total glycerol concentrations were lower in CC, compared to N, while TAG and FFA did not differ among the groups. However, the ratio of the FFA content to the TAG content was (tendency) higher in CC compared to N (FFA/TAG *p* = 0.079), indicating a relatively higher proportion of circulating FFA in the cachectic patients.

**Table 3:** Plasma lipid profile.

	***N***	**WSC**	**CC**	***p***
Total cholesterol (mg/dL)	206.0 [178.3; 237.5] (*n* = 24)	195.0 [168.0; 246.3] (*n* = 16)	179.0 [135.0; 235.0] (*n* = 23)	0.372
LDL (mg/dL)	109.0 [92.0; 133.0] (*n* = 19)	117.5 [79.5; 139.5] (*n* = 10)	95.0 [71.5; 132.0] (*n* = 17)	0.584
HHDL (mg/dL)	36.0 [32.0; 42.0] (*n* = 19)	45.0 [37.0; 56.0] (*n* = 14)	33.0 [30.0; 41.3]^#^ (*n* = 20)	**0.048**
TAG (mg/dL)	164.5 [99.3; 228.8.] (*n* = 24)	115.0 [74.3; 158.3] (*n* = 16)	96.0 [72.0; 130.0] (*n* = 23)	0.138
Glycerol (mg/ml)	0.014 [0.006; 0.022] (*n* = 25)	0.003 [0.001; 0.017] (*n* = 15)	0.004 [0.003; 0.009]^*^ (*n* = 22)	**0.013**
FFA (mg/dL)	0.95 [0.791; 1.18] (*n* = 25)	0.95 [0.691; 1.27] (*n* = 15)	1.09 [0.890; 1.41] (*n* = 22)	0.349
FFA/TAG (%)	0.006 [0.004; 0.009] (*n* = 24)	0.008 [0.006; 0.010] (*n* = 15)	0.011 [0.007; 0.015] (*n* = 22)	0.080
FFA/Glycerol (%)	70.0 [38.22; 131] (*n* = 25)	232 [41.1; 446] (*n* = 15)	179 [99.6; 254] (*n* = 22)	0.097

*Data are expressed as median [1st quartile; 3rd quartile], p, significance level Kruskal-Wallis test*.

*TAG, triacylglycerol; LDL, low density lipoprotein; HDL, high-density lipoprotein; FFA, free fatty acid*.

* *Significant difference vs. N (p < 0.05)*.

# *Significant difference vs. WSC (p < 0.05)*.

*p < 0.05 are highlighted in bold*.

### Cancer Cachexia Is Associated With Qualitative Changes in Plasma Fatty Acid Profile

**Figure 1:**
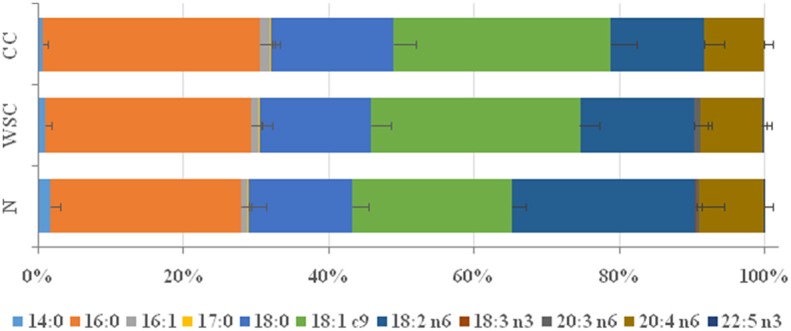
Plasma fatty acid profile in percentage. Data are expressed as median [1st quartile; 3rd quartile]. N, control; WSC, weight stable cancer; CC, cachectic cancer.

Among the types of fatty acids identified in the plasma of patients (Figure 1 and Table 4) changes in the percentage of different SFA were measured among the studied groups. The percentage of SFA palmitic acid (16:0) was higher (*p* = 0.006) in CC and WSC (27.98% and 27.83%) compared to N (24.1%), while stearic acid (18:0) content was only higher in CC (15.37%), compared to N (13.25%; *p* = 0.025). Myristic acid (14:0) was the only SFA whose abundance decreased (*p* = 0.019) in CC (0%) compared to *N* (1.74%). Regarding the unsaturated fatty acids, the presence of monounsaturated (MUFA) oleic acid (18:1n9) was significantly higher (*p* < 0.001) in the CC and WSC groups (28.55 and 27.39%), compared to N (20.56%). In contrast, the percentage of PUFAs was reduced in the same groups. The percentage of linoleic acid (LA; 18:2n6) was lower (*p* < 0.001) in CC and WSC (12.19 and 15.63%) compared to N (21.59%), and alpha linoleic acid (ALA; 18:3n3) was not detected (0%; *p* < 0.001) in both cancer groups. The percentage of the PUFAs gamma dihomo linoleic acid (DGLA, 20:3n6) and eicosapentaenoic acid (DPA 22:5n3) was decreased (*p* < 0.05) only in CC, compared to WSC. Arachidonic acid, a classic precursor for pro-inflammatory lipid mediators was not altered among the groups.

**Table 4:** Percentage of fatty acids profile in plasma.

**%**	***N* (*n* = 14)**	**WSC (*n* = 14)**	**CC (*n* = 14)**	***p***
14:0	1.74 [0.48; 2.8]	1.00 [0; 1.52]	0 [0; 1.21]^*^	**0.019**
16:0	24.10 [21.43; 26.41]	27.83 [26.98; 28.63]^*^	27.98 [26.02; 30.89]^*^	**0.006**
16:1	0.70 [0.35; 1.09]	1.08 [0.59; 1.43]	1.20 [0.79; 1.77]	0.065
17:0	0.08 [0; 0.22]	0.16 [0; 0.28]	0 [0; 0.12]	0.533
18:0	13.25 [11.9; 15.29]	13.93 [12.97; 17.0]	15.37 [14.01; 18.8]^*^	**0.025**
18:1c9	20.56 [19.4; 21.4]	27.39 [26.71; 29.90]^*^	28.55 [25.66; 29.83]^*^	**<0.001**
18:2n6	21.59 [21.24; 25.61]	15.63 [13.73; 16.50]^*^	12.19 [10.40; 13.67]^*^	**<0.001**
18:3n3	0.10 [0.06; 0.21]	0 [0; 0]^*^	0 [0; 0]^*^	**<0.001**
20:3n6	0.15 [0; 0.41]	0.35 [0; 1.78]	0 [0; 0.12]^#^	**0.034**
20:4n6	8.62 [7.92; 9.01]	8.50 [8.01; 8.88]	7.73 [6.99; 8.42]	0.146
22:5n3	0.04 [0; 0.09]	0.06 [0; 0.27]	0 [0; 0]^#^	**0.005**

*Data are expressed as median [1st quartile; 3rd quartile], p, significance level Kruskal-Wallis test. vs N*.

* *Significant difference (p < 0.05)*.

# *Significant difference vs. WSC (p < 0.05)*.

*p < 0.05 are highlighted in bold*.

### Quantitative Changes Saturated/Unsaturated of Fatty Acid Families in Cancer Cachexia

**Figure 2:**
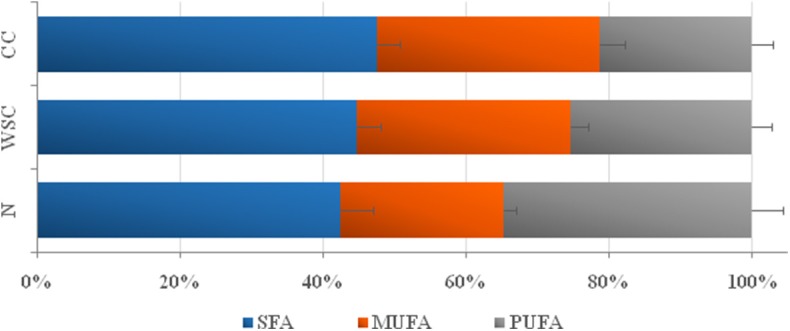
Plasma relative percentage of Saturated, Mono and Polyunsaturated free-fatty acids. Data are expressed as median [1st quartile; 3rd quartile]. SFA, saturated fatty acids (groupings of fatty acids 14:0, 16:0, 17:0, and 18:0); MUFA, monounsaturated fatty acids (groupings of fatty acids 16:1, 18: 1C9); PUFA: polyunsaturated fatty acids (groupings of fatty acids 18:2n6, 18:3n3, 20:n6, 20:4n6, and 22:5n3); n-3, omega-3 PUFA (groupings of fatty acids 18:3n3 and 22:5n3); n-6, omega-6 PUFA (groups of fatty acids 18:2n6, 20:n6 and 20:4n6).

Considering the groups of fatty acids, we found the proportion of SFA to be higher in CC compared to N, while MUFA content was higher in both CC and WSC in relation to N. Overall PUFA plasma content (n-3 and n-6) was lower in both cancer groups and also, the percentage of n-6 PUFA decreased in CC and WSC (p <0.001). Only the plasma content of n-3 PUFAs, known for their anti-inflammatory properties, was lower in CC as compared to the other groups. In general, our results indicate a shift from PUFAs, especially n-3 PUFAs, to a more saturated fatty acid profile in the plasma of cachectic patients, what may be related with the release or retention of these fatty acids by various tissues (Figure 2 and Table 5).

**Table 5:** Plasma relative percentage of Saturated, Mono and Polyunsaturated fatty acids.

**%**	***N* (*n* = 14)**	**WSC (*n* = 14)**	**CC (*n* = 14)**	***p***
SFA	39.67 [36.2; 42.59]	43.56 [40.71; 46.61]	44.2 [42.64; 47.46]^*^	**0.005**
MUFA	21.13 [20.13; 23.09]	28.44 [27.79; 30.68]^*^	29.85 [27.41; 30.88]^*^	**<0.001**
PUFA (n-3 + n-6)	32.14 [28.81; 34.89]	24.64 [22.37; 25.83]^*^	20.8 [17.29; 21.60]^*^	**<0.001**
PUFA n-3	0.13 [0.09; 0.28]	0.06 [0; 0.27]	0 [0; 0]^*^^#^	**<0.001**
PUFA n-6	32.04 [28.78; 34.62]	24.52 [21.84; 25.83]^*^	20.8 [17.29; 21.60]^*^	**<0.001**

*Data are expressed as median [1st quartile; 3rd quartile], p, Significance level Kruskal-Wallis test*.

*SFA, saturated fatty acids (groupings of fatty acids 14:0, 16:0, 17:0, and 18:0); MUFA, monounsaturated fatty acids (groupings of fatty acids 16:1, 18: 1C9); PUFA, polyunsaturated fatty acids (groupings of fatty acids 18:2n6, 18:3n3, 20:n6, 20:4n6, and 22:5n3); n-3, omega-3 PUFA (groupings of fatty acids 18:3n3 and 22:5n3); n-6, omega-6 PUFA (groups of fatty acids 18:2n6, 20:n6, and 20:4n6) vs. N*.

* *Significant difference (p < 0.05)*.

# *Significant difference vs. WSC (p < 0.05)*.

*p < 0.05 are highlighted in bold*.

### Increase of Circulating Pro-inflammatory Cytokines in Cancer Cachexia

The cytokine concentrations (Table 6) were measured in plasma, and IL-6, TNF-α and IL-8 were augmented in CC as compared to the control group N (*p* < 0.05), and IL-8 content was also higher in WSC compared to *N* (*p* < 0.05). CCL-2 and IFN-γ levels showed no differences among the groups. Anti-inflammatory factors such as interleukin-1-receptor agonist or IL-10 were not altered.

**Table 6:** Plasma inflammatory cytokines concentration.

**pg/ml**	***N***	**WSC**	**CC**	***p***
CCL-2	277 [239; 353] (*n* = 22)	267 [209; 401] (*n* = 16)	318 [265; 449] (*n* = 21)	0.238
IL-6	0.00 [0; 0.62] (*n* = 22)	1.18 [0.01; 2.53] (*n* = 16)	2.49 [0.85; 9.09]^*^ (*n* = 21)	**<0.001**
IFN-γ	1.28 [0.51; 1.91] (*n* = 20)	1.70 [0.73; 3.05] (*n* = 16)	1.48 [0.43; 3.37] (*n* = 20)	0.418
TNF-α	5.04 [3.81; 6.15] (*n* = 22)	6.80 [4.82; 9.68] (*n* = 16)	7.52 [5.71; 10.2]^*^ (*n* = 21)	**0.018**
IL-10	0.02 [0.01; 0.07] (*n* = 22)	0.05 [0.01; 0.49] (*n* = 16)	0.07 [0.04; 0.21] (*n* = 21)	0.091
IL-8	0.6 [0.4; 3.4] (*n* = 22)	5.8 [3.5; 7.2]^*^ (*n* = 16)	21.1 [5.1; 44.6]^*^ (*n* = 21)	**<0.001**
IL-1ra	0.22 [0.15; 0.28] (*n* = 22)	0.25 [0.18; 0.51] (*n* = 16)	0.31 [0.22; 0.41] (*n* = 21)	0.120

*Data are expressed as median [1st quartile; 3rd quartile], p, significance level Kruskal-Wallis test*.

*CCL2, monocyte chemoattractant protein; IL-6, interleukin-6; IFN-γ, interferon gamma; TNF-α, Tumor necrosis factor-alpha; IL-10, interleukin-10; IL-8, interleukin-8; IL-1ra, receptor antagonist of interleukin-1*.

* *Significant difference vs. N (p < 0.05)*.

*p < 0.05 are highlighted in bold*.

### Correlation Between Fatty Acid and Cytokines

We assessed the correlation of the fatty acid species and protein expression of pro and anti-inflammatory cytokines in plasma. First fatty acids were grouped according to their chemical classification. In the CC group there was a significant positive correlation between the SFA vs. IL-1ra (*r* = 0.653/*p*=0.039) and a significant negative correlation between PUFA-n3 vs. TNF-α (*r* = −0.800/*p* = 0.024) in the control group (Table 7). The correlation between individual fatty acids and cytokine concentrations revealed a negative correlation in the control group for heptadecanoic acid 17:0 vs. IL-6 (*r* = −0.890/*p* = 0.012) and vs. IL-1ra (*r* = −0.930/*p* = 0.009). In CC, stearic acid 18:0 was positively correlated with pro-inflammatory IL-8 (*r* = 0.764/*p* = 0.016) and CCL2 (*r* = 0.664/*p* = 0.036). The omega-6 fatty acid DGLA 20:3 n6 was positively correlated with IFN-Gamma (*r* = 0.687/*p* = 0.039) and IL-1ra (*r* = 0.648/*p* = 0.041) in CC, while in N we found a negative correlation of DGLA with IL-10 (*r* = −0.738/*p* = 0.037) and TNF-α (*r* = −0.831/*p* = 0.019). In the group of omega-3 fatty acids we only found a negative correlation of DPA 25:5n3vs TNF-α (*r* = 0.730/*p* = 0.039) in the control group. In the WSC group there was no correlation between cytokines and individual or grouped fatty acids (Table 7).

**Table 7:** Correlation of the percentage of fatty acids in plasma and protein expression of inflammatory markers of cachectic cancer patients.

			***N***	**WSC**	**CC**
SFA	*VS*	IL-1ra			0.653
					0.039
PUFA-n3	*VS*	TNF-α	-0.800		
			0.024		
Heptadecanoic (17:0)	*VS*	IL-1ra	-0.930		
			0.009		
Heptadecanoic (17:0)	*VS*	IL-6	-0.890		
			0.012		
Stearic (18:0)	*VS*	IL-8			0.764
					0.016
Stearic (18:0)	*VS*	CCL2			0.664
					0.036
DGLA (20:3 n6)	*VS*	IL-1ra			0.648
					0.041
DGLA (20:3 n6)	*VS*	IL-10	−0.738		
			0.037		
DGLA (20:3 n6)	*VS*	TNF-α	-0.831		
			0.019		
DGLA (20:3 n6)	*VS*	IFN-g			0.687
					0.039
DPA (22:5 n3)	*VS*	TNF-α	−0.730		
			0.039		

*Values of Spearman Correlation Coefficient (r) and significance level (p). r; p - data highlighted in blue r > 0.800; data highlighted in red p < 0.05*.

*Sample size (n). N (n = 8–9); WSC (n = 12); CC (n = 10–11)*.

## Discussion

Cancer cachexia is widely described and recognized as a chronic systemic inflammatory syndrome, promoting profound metabolic chaos; however, despite all the basic and clinical research efforts, the precise origin of cachexia remains unknown (2). Different types of fatty acids and their metabolites have been described as important mediators of chronic systemic inflammation, both acting as promoters or active players in the establishment or resolution of inflammation (33, 34). In the context of cancer, but not cachexia, lipid profile was the focus of several publications (35–37). Our group as one of few was able to detect quantitative differences of individual fatty acids in cachectic patients. Cachexia was associated with a significant reduction in body mass and body mass index accompanied by a decrease in overall quality of life, with the presence of anorexia measured by the QLQ-C30 and FAACT questionnaires. These findings corroborate several publications that investigated the quality of life of cachectic cancer patients (38, 39). Other characteristics of cachexia proposed by Evans et al. (1) such as anemia and hypoalbuminemia were also confirmed. CC exhibited hemoglobin concentrations below the reference value of 12 g/dL (11.2 [8.3; 12.1] g/dL) and although serum albumin levels in all groups of the study population were above the reference value of 3.2 g/dL, contents in CC were significantly lower than those of N. Systemic inflammation during cachexia is determined by serum C-reactive protein concentrations above 5 mg/L and we confirmed the inflammatory status of the cachectic cancer patients, since CRP serum levels in CC (11.5 [6.3, 12.7] mg/L) were about 10-fold higher than in the control group (1.15 [0.5; 3.4] mg/L). Hence, higher values of the CRP/albumin ratio in the CRP pointed to a relatively lower protein status in cancer cachexia albeit albumin concentrations were not below the reference value. Although the liver secretes an augmented concentration of acute-phase proteins in response to the presence of the extra-hepatic tumor, the albumin synthesis rate remains relatively constant (40). The reason for the reduced albumin concentrations in cancer cachexia seems to be tumor requirements. Albumin and other plasma proteins accumulate in the tumor tissue and are used as energy substrates and for *de novo* protein synthesis (41). The systemic inflammation observed in cancer cachexia results very likely from the interactions between tumor and host (42). Anatomical tumor site was not associated with cachexia and patients with the same tumor location may or may not be affected by the syndrome (Table 2). Therefore, the host's response to the presence of the tumor may be a more conceivable factor in the installation and maintenance of cachexia. The tumor induces alterations in energy metabolism (43, 44) and is capable of secreting numerous inflammatory mediators (45–47). We found the pattern of cytokine secretion to be different when comparing the tumors from cachectic and weight stable colorectal cancer patients (42). Inflammatory cytokines were shown to enhance lipolysis and fatty acid release from the adipose tissue or decreased lipogenesis in experimental cachexia models (48). It has been already described that in cachexia there is a deficit in the uptake of FFA and TAG from the circulation by peripheral tissues, what causes changes in circulating lipid profile (35, 49). Our results did not show alterations in total LDL, cholesterol and TAG, but in CC, even though not statistically significant, TAG blood levels were lower than in the other groups (Table 3). The total glycerol content was significantly increased, without changes in FFA concentration in CC group. However, when we analyzed the ratio of FFA/TAG, as well as the ratio of FFA/Glycerol we observed a relatively higher rate of FFA (*p* < 0.079 and 0.097, respectively) in the cachectic group. In an experimental model of cachexia associated with colon cancer, Tsoli et al. reported a similar decrease in the total content of TAG, with concomitant increase in FFA concentration (50). The increased FFA release is a result of enhanced lipolysis induced by pro-inflammatory cytokines from both blood cells and tissue itself (51). Silverio et al. reported increased lipolytic enzyme expression in cachectic patients (52). We have presently found changes in total plasma fatty acid profile such as an increase in the SFA palmitic acid (16:0) in both cancer groups, which may not only be due to increased lipolysis in WAT but also a consequence of increased synthesis in the tumor. It was already described that tumors exhibit a high expression of fatty acid synthase FASN which catalyses palmitate *de novo* synthesis (53). Stearoyl-CoA-desaturase expression was also shown to be augmented in breast cancer tissue leading to the release of C18:1 (54). Hence, also the higher relative percentage of MUFA oleic acid (18:1n9) observed in the cancer groups could be a result of the tumor burden. The dietary PUFAs C18:3n3 and C18:2n6 were lower in both cancer groups probably due to the consumption of less overall food or a shift in food choices as indicated by the altered anorexia score in these groups. Stearic acid (C18:0) content was exclusively higher in association with cachexia, it is yet to discover if the C18:0 proportion raises with ongoing adipose tissue depletion or if FA elongation via Elongase 6 is altered in cancer cachexia. Either way there is abundant evidence showing that SFA are potent activators of the pro-inflammatory Toll-like receptor family (TLRs) cascade, leading to increased production of cytokines via NFκB, also in cachexia (10, 55–57). In fact, in CC, SFA stearic acid (18:0) was positively correlated with the NF-κB targets CCL-2 and IL-8 and IL-6. We previously reported increased gene expression of the NF-κB p65 subunit and its pro-inflammatory target genes (TNF-α, IL-1β, and CCL2), and the gene expression of the regulatory protein NF-κB complex, IκB-α in cachectic patients (58). The binding of fatty acids to the TLR-4 receptor culminates in the release of the NF-κB dimer to exert its function in the induction of the expression of target genes as CCL2, IL-6, IL-8 and others (59). Batista et al. found increased plasma concentrations of IL-6 in cancer cachectic patients corresponding to a higher gene expression of that cytokine in SAT (12). de Matos-Neto et al. further demonstrated alterations of cytokine expression in SAT and in the tumor of cachectic patients (42). Considering the correlations between SFA and NF-κB targets in plasma of patients in the cachectic group of our study, we hypothesize a role for this pathway in the syndrome, acting as a contributor to the induction and maintenance of systemic inflammation.

In contrast to increased levels of SFAs our results indicate that unsaturated FA—mainly the n-3 FAs—are the also affected during cancer cachexia, since CC showed reduced percentage of such fatty acids, as compared to WSC. In the control group n-3 PUFAs were negatively correlated with TNF-α, pointing to their protective properties under physiologic conditions (21). The relative percentage of circulating n-6 PUFAs was reduced in CC, compared to N, while the n-3 PUFAs were absent in the CC group, consequently increasing the n-6/n-3 ratio.

The higher the ratio of n-6/n-3 FA the higher the risk of chronic diseases such as cancer, inflammation or cardiovascular diseases (60). The longer n-6 and n-3 fatty acids DGLA and DPA were diminished in CC, solely. This is in concordance with other studies describing a positive correlation between the times to death of advanced 118 cancer patients and lower amounts of both n3 and n6 FAs (23, 61). DGLA has anti-inflammatory properties because it can be converted to anti-inflammatory lipid mediators such as 1 series prostaglandins (PGD1, PGE1) and 15-HETE (62). These anti-inflammatory compounds were shown to suppress chronic inflammation, development of atherosclerotic plaques or growth and differentiation of tumor cells (62). In this anti-inflammatory context we found a positive correlation between DGLA and the anti-inflammatory cytokine IL-1rα in the CC group. In another CRC case and control study, Hodge et al. demonstrated that DPA C22:5n3 is inversely associated with CRC risk though absolute DPA C22:5n3 levels did not differ between cancer cases and controls thus implying a protective role of DPA. However, cachexia was not considered in the research (63). *In vitro* studies, DPA down regulated IL-6 in macrophages, inhibited TNF-α, improved cell membrane integrity and DPA–supplementation *in vivo* increased the concentration of EPA and DHA (64, 65). Thus, the lower plasma levels of C20:3n6 and C22:5n3 in cachectic patients of this study might further contribute to the inflammatory status. Studies that specifically investigate the circulating fatty acid profile of cancer cachexia patients are rare with most previous work concentrating on comparisons between cancer cases and controls. Hence the present study points out valuable ideas how changes in the FA profile could be a consequence of cancer cachexia and at the same time contributing factors to its progression. The present study evaluated FA in total plasma and not solely FFA, which is a limitation of the study, since it is not clear to what extent these fatty acids are unbound and possess signaling capacity. However, other publications showed that the total plasma fatty acid profile was comparable to the FFA profile (66) and it is assumed that the overall plasma fatty acid profile may also account for the inflammatory potential.

The relationship between FFA and cytokine production must nevertheless, be the target of future studies as to validate the hypothesis we present.

## Conclusion

This study establishes a positive correlation between lipid and pro-inflammatory mediators in human cancer cachexia. Systemic inflammation and changes in the fatty acids profile observed in CC suggest that the alterations of the relative proportion of circulating FA may play a role in the development and maintenance of inflammatory processes in cachexia. Further experiments are now needed to clarify to what extend the adipose tissue lipolysis and tumor metabolism modify the fatty acid profile and which particular inflammatory pathways are subsequently activated by FA.

## Data Availability Statement

The datasets analyzed in this article are not publicly available. Requests to access the datasets should be directed to dariccardi@hotmail.com.

## Ethics Statement

The studies involving human participants were reviewed and approved by Ethics Committee on Research involving Human Subjects of the Biomedical Sciences Institute/University of São Paulo and Human Ethics Committee of the University Hospital/University of São Paulo. The patients/participants provided their written informed consent to participate in this study.

## Author Contributions

DR: conception and design of the study, recruitment of patients, collection of samples, protein expression and FFA profile experiments, statistical analysis, and manuscript writing. RN: recruitment of patients, collection of samples, analysis of questionnaires and biochemical analysis, and manuscript writing. EM-N and MA: collection of samples, intellectual support, and review of the manuscript. RGC: collection of samples, intellectual support, analysis of questionnaires, and biochemical analysis. JL: collection of samples, intellectual support, biochemical analyzes, and review of the manuscript. KR: recruitment of patients, collection of samples, analysis of questionnaires and biochemical analyzes, and review of the manuscript. RGFC: recruitment of patients, collection of samples, biochemical analyzes, intellectual support, and review of the manuscript. FT, JO, LM, and PA: recruitment of patients, collection of samples, and intellectual support. AC: FFA profile analysis, statistical analysis, intellectual support, and review of the manuscript. AL: intellectual support and review of the manuscript. MS: conception, design and supervision of the study, and manuscript writing.

### Conflict of Interest

The authors declare that the research was conducted in the absence of any commercial or financial relationships that could be construed as a potential conflict of interest.
